# Brivaracetam Combined With Topiramate at Low Doses Alleviates Neurobehavioral Deficits and Oxidative Stress in a Chemoconvulsant Kindled Seizure Model

**DOI:** 10.1155/bn/8037864

**Published:** 2025-12-11

**Authors:** Khaled Ahmed Saghir, Waseem Ashraf, Rana Muhammad Zahid Mushtaq, Faleh Alqahtani, Imran Imran

**Affiliations:** ^1^ Department of Pharmacology, Faculty of Pharmacy, Bahauddin Zakariya University, Multan, Pakistan, bzu.edu.pk; ^2^ Institute for Regeneration and Repair, Edinburgh Medical School, The University of Edinburgh, Edinburgh, UK, ed.ac.uk; ^3^ Department of Pharmacology and Toxicology, College of Pharmacy, King Saud University, Riyadh, Saudi Arabia, ksu.edu.sa

**Keywords:** brivaracetam, cognitive impairment, depressive-like behavior, epilepsy, oxidative stress, PTZ kindling, topiramate

## Abstract

Epilepsy is a long‐lasting neurological condition often associated with cognitive and behavioral comorbidities, such as anxiety, depression, and memory deficits. This study examined the therapeutic effect of topiramate (TPM) and brivaracetam (BRV), both separately and combined, using pentylenetetrazole‐kindled mice. Seizure severity was visually observed during the kindling process. After that, mice underwent a series of behavioral tests to evaluate anxiety, depression, and memory performance. Subsequently, neurochemical analyses were performed to assess cholinergic activity and oxidative stress markers. The TPM + BRV group showed significantly attenuated seizure progression during all PTZ doses (*p* < 0.001), with an 88.4% reduction in seizure scores compared to monotherapies. PTZ‐kindled mice showed marked behavioral impairments and biochemical imbalances, including elevated oxidative stress and acetylcholinesterase activity. While monotherapy with BRV or TPM displayed partial improvements, combined therapy provided significantly greater effects, enhancing central explorations (152% and 259.6%), sociability (195.2%), memory retention (508.4% for discrimination index and 463.9% for aversive awareness), and reducing depressive‐like behaviors (52.7%–73.7%). Biochemically, the combined treatment restored antioxidant enzyme levels (SOD, CAT, and GPx) by 45%–70% and significantly lowered MDA levels (70.7%) and restored SOD activity (220.9%). These findings suggest that low‐dose rational polytherapy with TPM and BRV may enhance seizure control and ameliorate associated neuropsychiatric and oxidative imbalance.

## 1. Introduction

Epilepsy, which affects over 70 million people globally, is a lifelong neurological disorder characterized by unprovoked and recurrent seizures [1]. However, the condition involves much more than just seizure episodes. Many epileptic patients also face behavioral and emotional challenges, such as anxiety, depression, and memory impairments, that significantly reduce quality of life and impair daily activities [2].

The pentylenetetrazole (PTZ) kindling model has gained significant attention because of its ability to simulate the progressive nature of epilepsy and its associated behavioral abnormalities more effectively than acute seizure models [3]. Unlike models that only replicate acute seizures, PTZ kindling allows assessment of the long‐term effects of repeated seizures and epilepsy‐related behavioral changes. Animals in this model not only display chronic seizure activity but also cognitive deficits alongside anxiety and depressive‐like behaviors [4]. The pathophysiology behind these impairments involves persistent oxidative stress, neuroinflammation, and impaired neurotransmitter homeostasis. These processes contribute to both structural and functional impairments in the hippocampus and prefrontal circuits critical for cognition and emotional regulation [5].

Although current antiseizure medications are relatively successful in controlling seizures in many patients, they often fail to address the emotional and cognitive symptoms. Some drugs, like valproic acid, phenytoin, and levetiracetam, may even worsen these issues [6]. This highlights the urgent need for treatments that not only control seizures but also support brain health and behavior. Rational polytherapy, which combines drugs with complementary mechanisms, offers a promising approach. It is aimed at increasing treatment effectiveness while minimizing side effects [7]. Topiramate (TPM) has known neuroprotective effects and enhances GABAergic inhibition [8]. Brivaracetam (BRV) targets a different receptor, the SV2A protein on neurotransmitter‐containing synaptic vesicles, which modulates neurotransmitter release with fewer behavioral side effects compared to levetiracetam [9]. Their combined use can synergistically modulate both the excitatory and inhibitory systems and reduce inflammatory and oxidative pathways.

To date, no published studies have assessed the combined effects of TPM and BRV in PTZ‐kindled mice with integrated behavioral and neurobiochemical endpoints. We focused on this gap and assessed anxiety‐like behaviors, depressive symptoms, and cognitive performance. At the same time, we measured markers of oxidative stress and enzymes involved in neurotransmitter regulation to gain a better understanding of the biochemical basis of any behavioral improvements. These multitargeted therapeutic strategies are aimed at treating not only seizures but also the broader mental and cognitive burdens associated with epilepsy.

## 2. Methods

### 2.1. Animals and Drugs

Forty‐eight male C57BL/6 mice (23 ± 2 g) were utilized and kept at the Faculty of Pharmacy, Animal Center, in controlled environmental conditions (12‐h light/dark cycle, 23^°^C ± 2^°^C) under free access to water and food. The Ethics Committee of the Department of Pharmacology, BZU, Multan, Pakistan (Approval No. 02/PHDL/S/23, 20/02/2024) permitted the study, where the study protocol was performed and conducted following the ARRIVE guidelines. The experiments were carried out from 8:00 a.m. to 4:00 p.m.

The dosages were chosen following previously published research studies. TPM (Hilton Pharma, Pakistan; 10 mg/kg) [10], BRV (Zhejiang Eazy Pharmchem, China; 10 mg/kg) [11], VPA (Sigma‐Aldrich, Germany; 150 mg/kg) [12], and PTZ (Sigma‐Aldrich, Germany; 40 mg/kg) [13] were dissolved in 0.9% normal saline (NS) solution. The 1‐NS mice were injected with NS (1 mL/kg). All drugs were prepared fresh and injected intraperitoneally.

### 2.2. Study Design

**Figure 1:**
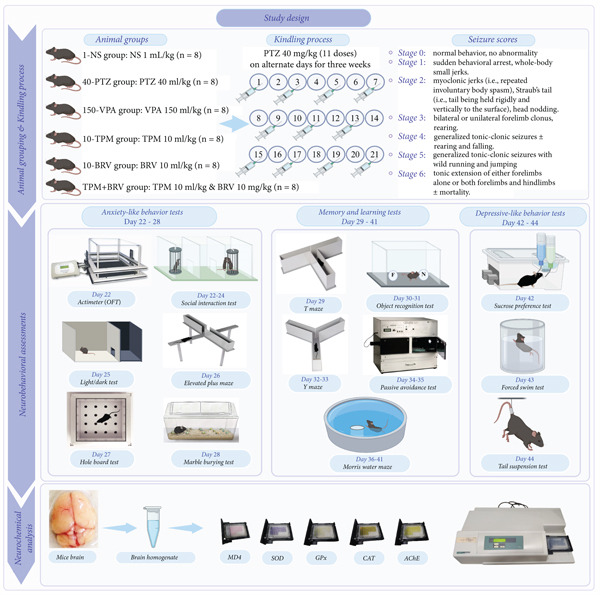
Schematic overview of the experimental protocol. All mice, except the 1‐NS group, were exposed to the kindling protocol and later assessed for neurobehavioral assessments and neurochemical analysis. This figure (QB28LRQGY, dated August 7, 2025) was designed with https://www.biorender.com.

Mice were randomly grouped into six groups: 1‐NS (NS, 1 mL/kg, *n* = 8), 150‐VPA (VPA 150 mg/kg, *n* = 8), 40‐PTZ (PTZ 40 mg/kg, *n* = 8), 10‐TPM (TPM 10 mg/kg, *n* = 8), 10‐BRV (BRV 10 mg/kg, *n* = 8), and TPM + BRV (TPM and BRV 10 mg/kg each, *n* = 8). All mice, except the 1‐NS group, underwent the kindling protocol and received PTZ (40 mg/kg) on alternate days for 3 weeks. Seizure severity was monitored individually for 30 min and scored using the modified Racine scale (Table 1) [14]. Mice were considered fully kindled upon exhibiting at least three consecutive Stage 4 seizures [13, 15]. Twenty‐four hours after the last PTZ dose, mice were subjected to neurobehavioral assessments and subsequently to neurochemical analysis. The experimental design is illustrated in Figure 1. The investigators who performed all neurobehavioral and neurobiochemical measures were blinded to treatment groups.

**Table 1:** Behavioral seizure stages of the modified Racine scale for seizure severity in observed PTZ‐kindled mice. The table describes categories of seizure severity, which are commonly observed in PTZ kindling models of epilepsy. Each stage represents rising neurological and motor activity, from no observable signs (Stage 0) to tonic extension with or without mortality (Stage 6). This scale was employed to assess the development and severity of seizures throughout the PTZ kindling process.

**Stage**	**Seizure description**
0	Normal behavior, no abnormality
1	Sudden behavioral arrest, whole‐body small jerks
2	Myoclonic jerks (i.e., repeated involuntary body spasms), Straub′s tail (i.e., tail being held rigidly and vertically to the surface), head nodding
3	Bilateral or unilateral forelimb clonus, rearing
4	Generalized tonic–clonic seizures with or without rearing and falling
5	Generalized tonic–clonic seizures with wild running and jumping
6	Tonic extension of either forelimbs alone or both forelimbs and hindlimbs, with or without mortality

### 2.3. Neurobehavioral Assessments

After 24 h from the last PTZ dose, all mice were subjected to neurobehavioral tests to assess psychiatric comorbidities associated with postkindling. Prior to each test, mice were acclimatized for 30–60 min in the behavioral room. The maze was thoroughly cleaned after each trial in every test. Preclinical neurobehavioral tests were categorized into anxiety‐like behavior tests (open field test [OFT], social interaction test [SIT], light/dark test [LDT], elevated plus maze [EPM], hole board test [HBT], and marble burying test [MBT]); memory and learning tests (passive avoidance test [PAT], T maze, object recognition test [ORT], Y maze, and Morris water maze [MWM]); and depressive‐like behavior tests (sucrose preference test [SPT], forced swim test [FST], and tail suspension test [TST]). All neurobehavioral tests were recorded by a Logitech camera and analyzed by a fully licensed 7.33 version of Any‐maze video‐tracking software, except MBT and SPT, which were manually noted.

### 2.4. Anxiety‐Like Behavior Tests

#### 2.4.1. OFT

Locomotor activity and anxiety‐like behavior following the kindling process were evaluated in the OFT. Mice were individually placed in the center of the open‐field maze (45 × 45 × 20 cm) assembled with 16 × 16 lower and upper infrared photo beam sensors (Panlab Harvard apparatus IR Actimeter, Spain). Each mouse freely explored the maze for 5 min, and olfactory cues were removed with 70% alcohol after each trial. The maze was divided into central and peripheral zones to predict the mouse′s anxiety from the preference zone. The total distance traveled, speed, vertical and horizontal activities (fast rearings and fast movements), time spent, and entries in the center were observed to measure anxiety levels between kindled and treated mice [16].

#### 2.4.2. SIT

The maze consisted of three rectangular chambers of equally sized (20 × 40 × 47 cm) separated by transparent Plexiglas walls with middle doors (5 × 3 cm). A spherical metal wire cage with vertical bars 0.5 cm apart, 11 cm in height, and 9 cm in diameter was positioned in both the right and left chambers [17]. The test was divided into a habituation period and two sessions named the social affiliation aspect test (sociability test) and the social novelty preference test, respectively. The mice were individually habituated in the middle chamber for 5 min, where it was closed and no animals were inside cages. After that, one mouse (Stranger 1) was placed in one cage while the other cage was empty. The doorways of the middle chamber were opened and the subject mice freely explored the whole maze for 5 min. In the second session, another mouse (Stranger 2) was placed in the other cage and all mice freely re‐explored both familiar and novel mice for 5 min. Both mice in cages were of the same sex and age as the subject mice. The time spent around each cage in the two sessions was noted to calculate the sociability index (SI) and social novelty preference to evaluate social interactions [18]. SI was calculated according to the formula:

SI=interaction time with Stranger 1−interaction time with empty cage/interaction time with Stranger 1+interaction time with empty cage.

Preference for social novelty was calculated as follows:

Preference of social novelty %=interaction time with Stranger 2/total interaction times with Stranger 12&Stranger ×100

Anxious mice naturally show fewer social interactions and less preference percentage for novel mice as compared to familiar mice.

#### 2.4.3. LDT

The light/dark maze consisted of two compartments, a light chamber (21 × 21 × 25 cm) and a dark chamber (20 × 40 × 40 cm) connected with a small opening door (7 × 6 cm). Mice were individually placed in the light chamber and allowed to freely explore the maze for 5 min. The time spent and entries in the light chamber were noted to evaluate the anxious behavior [19]. Anxious mice prefer to enter and spend more time in a dark chamber than in a light chamber.

#### 2.4.4. EPM

EPM is a plus‐shaped maze that is elevated 45 cm above the ground with a center zone (5 × 5 cm) surrounded by two opposed closed arms (25 × 5 cm, height 15 cm) and two opposed open arms (25 × 5 cm, height 3 mm). Mice were individually placed in the center zone, and the maze was explored for 5 min. To evaluate anxiogenic behavior, the time spent and entries in open arms were noted [20]. Anxious mice prefer to enter and spend more time in closed arms than in open arms.

#### 2.4.5. HBT

Mice were individually placed in the hole board maze (40 × 40 × 25 cm) containing 16 central holes (2.5 cm) in which mice could dip their heads. After 5 min of free exploration, latency to the first head dip and number of head dippings were noted to evaluate the anxiety status of mice [21]. Anxious mice show less neophiliac activity and lack the desire to dip their heads into the board′s holes.

#### 2.4.6. MBT

Transparent cages (28 × 45 × 14 cm) containing sawdust were used to perform MBT. Fifteen marbles were distributed equally on the surface of the sawdust in a 3 × 5 pattern. Mice were placed separately in the cages and allowed to explore the marbles for 30 min. After that, unburied marbles were counted. The marble was considered buried if it was ≤ 1/3 visible on the sawdust [22]. Anxious mice (both male and female) behave naturally to bury more marbles in sawdust with their noses and forepaws.

### 2.5. Memory and Learning Tests

#### 2.5.1. T Maze

The T maze test evaluates spatial working memory in a less aversive and friendlier environment. The principle of the T maze relies on the natural tendency of rodents to roam toward a new arm rather than a familiar arm, which results in altered behavior [23]. The T maze consists of a vertical arm (30 × 10 × 20 cm) positioned at 90° to two horizontal arms forming a T‐shaped structure. Mice were individually placed in the maze′s center and allowed free exploration to all arms for 5 min [17]. Numbers and sequences of arm entries were noted to calculate SA (consecutive entries into all three arms) according to the formula:

SA %=no.of alternations/total arm entries−2×100

Cognitive deficit mice show less altered behavior and a low % age SA, indicating impaired working memory and hippocampal dysfunction.

#### 2.5.2. ORT

This test was conducted to assess memory and learning, especially object recognition memory [24]. An open maze (40 × 40 × 38 cm) containing two objects was used for this test. The objects were arranged symmetrically at opposite sides, facing each other, at a 20‐cm distance, 10 and 21 cm away from the center and corner, respectively [25]. The test consisted of two sessions: a training session and a testing session. In the training session, mice were individually placed in the maze containing two identical objects and freely explored both objects for 5 min. Twenty‐four hours later, the testing session was performed, where one object was replaced by a novel object. All mice were re‐explored freely to familiar and novel objects for 5 min. The exploration time of both objects and the discrimination index (DI) were noted [24]. The DI, which measures the animal′s ability to differentiate between two dissimilar objects simultaneously present, was calculated as follows:

DI=novel object exploration time−familiar object exploration time/novel object exploration time+familiar object exploration time

Cognitive deficit mice show less exploration time for novel objects and a low DI.

#### 2.5.3. Y Maze

This test was conducted to assess spatial working and reference memory [26]. The Y maze contained three equal‐sized arms (40 × 8 × 15 cm) positioned at 120°, forming a Y‐shaped structure. The test consisted of two sessions: a training session and a testing session. In the training session, mice were individually placed in the maze where one arm was closed (novel arm) and mice freely explored two arms for 5 min. Twenty‐four hours later, a testing session was performed where all arms were opened. All mice re‐explored freely to all maze′s arms for 5 min [26]. The time spent in all arms was noted to calculate novelty preference according to the formula:

Novelty preference %=time in novel arm/total times in all arms×100

Cognitive mice naturally show less novelty preference and low‐interest behavior toward novel arms indicating impairment in spatial memory and hippocampal dysfunction.

#### 2.5.4. PAT

The passive avoidance maze (Gemini avoidance system, San Diego Instruments, United States) contains two equally sized chambers: light and dark (9.5 × 8 × 8 cm), connected by an internal door. The test comprised a single acquisition trial and two retention trials and was attained by Gemini Software Version 1.0.4. Mice were individually placed and acclimatized in the light chamber for 30 s; then, the internal door was opened and mice freely explored both chambers for 150 s. As soon as the mice entered the dark chamber, the internal door was closed and an electric shock (aversive stimulus) of 0.3 mA for 2 s was applied in the acquisition trial; then, after 30 s, the mice were removed and returned to the home cage [25]. One hour later, the first retention trial was performed, and the second retention trial was done after 24 h. In both retention trials, the internal door was opened after 20 s of acclimation and the mice freely explored both chambers for 300 s and no shock was applied. The latency to enter the dark chamber was noted for all trials to evaluate both short‐term and long‐term memory recognition. Cognitively impaired mice are more likely to show shorter latency to enter the aversive stimulus chamber.

#### 2.5.5. MWM

This test was conducted to assess long‐term memory and learning function [27]. In MWM, a circular tank (100 × 60 cm) was filled with nontoxic cloudy dyed water, equally partitioned into four quadrants (SW, SE, NW, and NE), and surrounded by different colored and shaped geometrical cues. Proximal cues were fixed on the inner side of the maze, while distal cues were located around the poles. The platform (12 × 12 cm) was situated in the center of the SW quadrant. The test was divided into two training days, three testing days, and one probe day, lasting 6 days continuously. Firstly, in the two training days, all mice were trained in three trials a day, where mice were individually placed in the maze and allowed to reach the visible platform within 120 s [28]. Once reached, mice were allowed to stay for 10 s. When mice failed to reach the platform, slight guidance and 20 s of staying on the platform were given to memorize orientation cues [29]. Subsequently, in the three testing days, the platform was submerged 2.5 cm below water. All mice were tested once a day (one trial different from other days) and allowed to reach the invisible platform within 120 s. The latency to reach the platform (escape latency) on the training and testing days was noted. Finally, on the probe day, the platform was removed, all mice swam for 120 s, and the time spent and visiting numbers to the targeted quadrant where the platform was previously placed were calculated [30]. Cognitive mice show low learning ability and impaired memory through long latency, less time spent, and fewer visiting numbers to the targeted quadrant.

### 2.6. Depressive‐Like Behavior Tests

#### 2.6.1. SPT

Generally, rodents have a natural preference for sweet solutions; therefore, the SPT was developed to evaluate anhedonia and depressive‐like behaviors [31]. After fasting for 12 h, all mice were individually provided one bottle filled with 100 mL of 2% sucrose solution and another bottle filled with 100 mL of tap water, with no access to food during the test. Twenty‐four hours later, the consumption of sucrose solution and water was noted to calculate % sucrose preference [32] according to the formula:

Sucrose preference%=sucrose solution intake water intake+sucrose solution intake×100

Depressed mice consume less sucrose solution and display a lower sucrose preference percentage compared to normal mice.

#### 2.6.2. FST

FST was conducted to assess depression and phenotypes related to antidepressants [33]. Mice were gently held by the tail and slowly placed in a transparent glass cylinder (30 × 20 cm) filled with 15 cm of water [34] at 22°C–23°C and allowed to swim for 4 min; then, the immobility time was noted to observe the depressive state [29].

#### 2.6.3. TST

TST was conducted to assess depression and behaviors related to antidepressants. All mice were individually suspended by their tails with adhesive tape for 6 min, and immobility time was noted to observe a depressive state [35].

### 2.7. Neurochemical Analysis

Immediately after completion of neurobehavior tests, mice (*n* = 4) were selected randomly for neurochemical analyses. To minimize distress, mice were deeply anesthetized with 5% isoflurane (*v*/*v* in synthetic air) delivered through a calibrated vaporizing system (Kent Scientific Corporation, United States). Anesthesia was maintained in the induction chamber (Word Precision Instruments, United States) for 1–2 min until observation of complete loss of reflexes. Euthanasia was performed by decapitation using a rodent guillotine, allowing for rapid brain removal. This procedure followed our lab′s established protocols and complied with institutional ethical standards [36]. The isolated brains were further processed for neurochemical processes, that is, homogenization and enzymatic assays. To assess markers of oxidative stress and neuroinflammation, different enzymatic assays were performed, such as malondialdehyde (MDA), superoxide dismutase (SOD), glutathione peroxidase (GPx), catalase (CAT), and acetylcholinesterase (AChE) assays. Standard (if necessary) and blank samples were simultaneously run in every test. Samples according to the microplate protocol were loaded in a 96‐well microplate in duplicates, and absorbances were noted on the microplate reader (SpectraMax 340 PC384 by Molecular Devices, California, United States). Protein contents were quantified according to Lowry′s protocol [37], and each result of assays was subsequently normalized based on this quantification. Using a subset of mice (*n* = 4 per group) due to resources and ethical restraints is considered a study limitation. However, this sample size was adequate to identify moderate to large effect sizes.

### 2.8. Brain Homogenization

Isolated brains of 0.3 g each were homogenized individually in 3‐mL phosphate buffer saline (10 *w*/*v*) of pH 7.4 (PBS, Solarbio, Life Sciences, China) and subsequently centrifuged for 10 min at 12,000 rpm and 4°C. The clear supernatants were stored at −40°C for further enzymatic assays [28].

### 2.9. MDA Assay

To estimate lipid peroxidation, brain homogenate (100 *μ*L) was mixed with a mixture in a 1:1 ratio of 15% trichloroacetic acid (TCA, Uni‐Chem Chemical Reagents) and 0.37% thiobarbituric acid (TBA [C_4_H_4_N_2_O_2_S], Uni‐Chem Chemical Reagents) and boiled at 100°C for 15 min. The light pink‐colored mixture was cooled at room temperature and centrifuged for 10 min at 3500 rpm and 4°C. Absorbance at 532 nm was noted and MDA levels were calculated and presented as nmol/mg of protein [38].

### 2.10. SOD Assay

The following chemicals were mixed with brain homogenate (50 *μ*L) to estimate SOD activity: 50 *μ*L of 50 mM sodium carbonate (Na_2_CO_3_, Sigma‐Aldrich, Germany), 20 *μ*L of 0.1 mM ethylene diamine tetra‐acetic acid (EDTA, Sigma‐Aldrich, Germany), and 40 *μ*L of 0.56 mM nitro blue tetrazolium (NBT, Molekula, United Kingdom). The reaction started when 40 *μ*L of 0.1 mM hydroxylamine chloride (HAC, Sigma‐Aldrich, Germany) was added to the mixture, resulting in a light purple color. Absorbances at 570 nm were noted for 45 min with 5‐min intervals, and % inhibition of SOD activities was calculated and presented as SOD units/milligram of protein [11].

### 2.11. GPx Assay

Mixtures of 20 *μ*L of 0.8 mM EDTA, 10 *μ*L of 10 mM sodium azide (Sigma‐Aldrich, Germany), 10 *μ*L of 2.5 mM hydrogen peroxide (H_2_O_2_, Sigma‐Aldrich, Germany), 20 *μ*L of 0.1 M reduced glutathione (C_10_H_17_N_3_O_6_S, Oakwood Chemical, United States), and 20 *μ*L of PBS were added to 20 *μ*L of brain homogenate and incubated for 15 min at 37°C. Forty microliters of 10% TCA was added to stop the reaction and the mixture was centrifuged for 5 min at 15,000 rpm and 4°C. Then, 30 *μ*L of 0.3 mM disodium hydrogen phosphate (Na_2_HPO_4_, Sigma‐Aldrich, Germany) and 70 *μ*L of 0.04% 5,5 ^′^‐dithio‐bis‐2‐nitrobenzoic acid (DTNB, Sigma‐Aldrich, Germany) were added to 100 *μ*L of supernatant of the reaction mixture which resulted in a light yellow color. Absorbance at 412 nm was noted, and GPx activity was calculated and presented as nmol/min/mg of protein [39].

### 2.12. CAT Assay

A mixture of 10 *μ*L of brain homogenate, 50 *μ*L of PBS, and 40 *μ*L of 0.2 M H_2_O_2_ was incubated for 90 s at 37°C, and then, 100 *μ*L of 5% potassium dichromate acetic acid was added to stop the reaction, and a blue‐colored mixture was obtained. After that, the reaction mixture was boiled for 15 min at 100°C, and the obtained light green–colored mixture was centrifuged for 5 min at 2500 rpm and 4°C. Absorbance at 570 nm was noted, and CAT activity was calculated and presented as *μ*mol/min/mg of protein [16].

### 2.13. AChE Assay

An amount of 40 *μ*L of brain homogenate, 138 *μ*L of PBS, and 20 *μ*L of 0.01 M DTNB were mixed, and the baseline absorbance for the mixture was noted at 412 nm. Then, 2 *μ*L of 0.075 M acetylthiocholine iodide (Sigma‐Aldrich, Germany) was added, resulting in a yellowish‐colored mixture. Absorbance changes at 412 nm were noted every 2 min up to 10 min, and AChE activity was calculated and presented as *μ*mol/min/mg of protein [20].

### 2.14. Data Analysis

The Shapiro–Wilk test and G∗Power were employed to assess data normality and sample size, respectively. Differences among groups in neurobehavioral and neurochemical tests were analyzed using one‐way ANOVA, while escape latency in PAT and MWM tests was analyzed by two‐way ANOVA, followed by post hoc Tukey′s multiple comparisons when significance was detected. Data were displayed as mean ± SD, and a *p* value of less than 0.05 was considered statistically significant. Statistical analyses were performed by GraphPad Prism Version 9.5.0 (San Diego, California, United States).

In order to estimate the treatment size effects, Cohen′s *d* was determined for pairwise comparison of the 40‐PTZ group with each treatment group using pooled standard deviations and calculated as

d=meantreatment−meanPTZSDpooled

where

SDpooled=SD2treatment+SD2PTZ2

Absolute effect sizes were considered to be very small (*d* = 0.01), small (*d* = 0.20), medium (*d* = 0.50), large (*d* = 0.80), and very large (*d* ≥ 1.30). Relative improvements (%) were calculated by using the following formula:

Relative improvements %=treatment mean−PTZ meanPTZ mean×100

Positive values represent improvement, while negative values demonstrate beneficial reduction.

## 3. Results

### 3.1. Seizure Score Analysis

**Figure 2:**
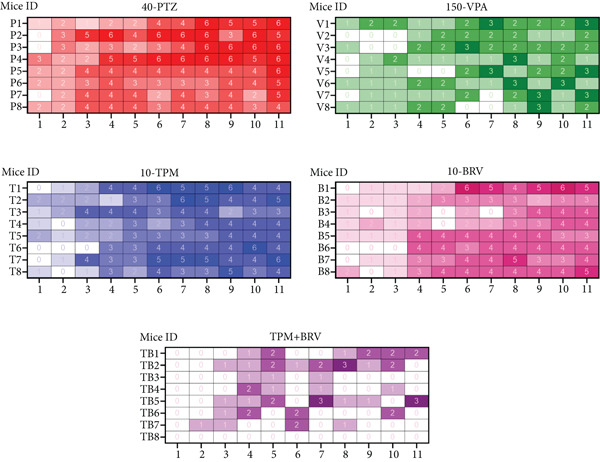
Visualization heatmap of seizure severity scores during the PTZ kindling process across experimental groups. Exposure to the PTZ control aggravated the seizures progressively, whereas VPA, TPM, and BRV monotherapies alleviated the attacks to different extents. The TPM + BRV group showed significantly attenuated seizure progression among all treatment groups.

Throughout the PTZ kindling process, seizure severity was assessed using the modified Racine scale. As shown in the heatmap (Figure 2), the 40‐PTZ group exhibited a gradual increase in seizure severity over time, indicating successful kindling in all mice. The 150‐VPA group showed a notable decrease in seizure severity, consistent with the documented anticonvulsant effect of VPA. Partial suppression was observed in both the TPM and BRV monotherapy groups. The most prominent attenuation was observed in the TPM + BRV group during the kindling process across all groups (88.4%, *d* = 4.79, very large reduction at the last PTZ dose), suggesting a potential synergistic effect in seizure management.

### 3.2. Treatment Effect on Neurobehavioral Assessments

Statistical details, including *F* and *p* values, effect sizes (Cohen′s *d*), and relative improvements (%) for all neurobehavioral and neurobiochemical tests across treatment groups, are summarized in Tables S1 and S2.

### 3.3. Anxiety‐Like Behavior Tests

#### 3.3.1. OFT

**Figure 3:**
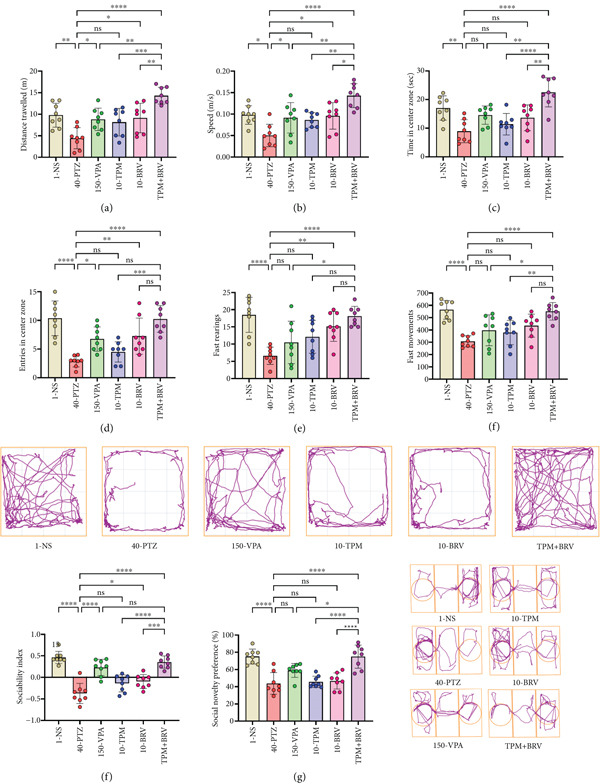
Treatment effect in (a) differences for distance traveled during 5 min, (b) speed in the maze, (c) time spent, (d) number of entries to the center zone, (e) fast rearings, and (f) fast movements for OFT. (g) Sociability index, (h) % preference of social novelty, and track plots for SIT. All data were displayed as mean ± SD (*n* = 8), and statistical significance was  ^∗^ *p* < 0.05,  ^∗∗^ *p* < 0.01,  ^∗∗∗^ *p* < 0.001, and  ^∗∗∗∗^ *p* < 0.0001.

Many parameters, including distance traveled, movement speed, central zone preference, and actimeter parameters, were noted in OFT to evaluate anxiety‐like behaviors for all groups. The 40‐PTZ mice showed reduced exploration (distance traveled: 4.423 ± 2.466 m; speed: 0.050 ± 0.026 m/s). TPM + BRV mice exhibited the highest activity (distance traveled: 14.319 ± 1.938 m; speed: 0.143 ± 0.028 m/s; *p* < 0.0001), representing a 223.8% increase in distance and a 186% increase in speed, with very large effect sizes (*d* = 4.72 and 3.45, respectively). VPA showed moderate improvements, while TPM and BRV monotherapies were less effective (Figure 3a,b).

Central zone preference, an anxiolytic behavior indicator, was evaluated by time spent and entries. 40‐PTZ mice remained thigmotaxic (time: 8.938 ± 4.029 s; entries: 2.850 ± 0.974), while TPM + BRV mice showed enhanced exploration (time: 22.513 ± 5.063 s; entries: 10.250 ± 2.315; *p* < 0.0001), indicating a 151.8% increase in time and a 259.6% increase in entries with large effect sizes (*d* = 3.02 and 3.87, respectively). VPA showed partial efficacy, while TPM and BRV alone did not significantly improve central zone behavior (Figure 3c,d).

Locomotor activities, including fast rearings and fast movements, were measured by the actimeter. 40‐PTZ mice showed reduced activity (fast rearings: 6.625 ± 2.504; fast movements: 307.625 ± 45.239). TPM + BRV‐treated mice showed full restoration of these behaviors (fast rearings: 18.125 ± 2.850; 173.6%; fast movements: 563.625 ± 75.683; 79.3%, *p* < 0.0001), with large effect sizes (*d* = 5.25 and 4.76, respectively). Other treatments showed minimal or no significant effects (Figure 3e,f).

#### 3.3.2. SIT

In the sociability test, mice were given the option to interact either with an unfamiliar mouse (Stranger 1) or with the empty cage side. Among all groups, 40‐PTZ mice showed impaired sociability (SI = −0.373 ± 0.234). TPM + BRV mice demonstrated a 195.2% improvement (SI = 0.355 ± 0.142, *p* < 0.0001), with a very large effect size (*d* = 3.56). VPA showed significant improvement, while TPM and BRV monotherapies were less effective (Figure 3g).

In the preference of social novelty test, mice were given the option to interact either with the previously familiar mouse (Stranger 1) or the novel mouse (Stranger 2). 40‐PTZ mice showed less interest in novel mice and spent more time with familiar mice, resulting in a reduced % preference of social novelty (43.854*%* ± 12.589*%*) among all groups. TPM + BRV‐treated mice demonstrated a 71% improvement (74.0.084 ± 7.992; *p* < 0.0001), with a large effect size (*d* = 2.73). VPA, TPM, and BRV monotherapies showed no significant improvement. TPM + BRV was significantly more effective than all other treatments (Figure 3h).

#### 3.3.3. LDT

**Figure 4:**
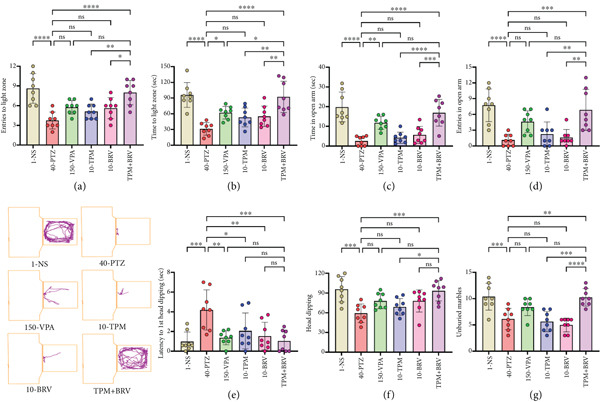
Treatment effect in (a) entries into the light chamber, (b) time spent in the light chamber for LDT, (c) time spent in open arms, (d) entries into open arms for EPM, (e) latency of first head dipping, (f) head dipping into holes for HBT, and (g) unburied marble in MBT. All data were displayed as mean ± SD (*n* = 8), and statistical significance was  ^∗^ *p* < 0.05,  ^∗∗^ *p* < 0.01,  ^∗∗∗^ *p* < 0.001, and  ^∗∗∗∗^ *p* < 0.0001.

Mice′s behavior against light and dark chambers was evaluated in LDT by measuring entries and time spent in the light chamber. Mice of the 40‐PTZ group showed reduced exploration (entries: 3.750 ± 1.282; time: 31.200 ± 11.941 s), indicating heightened anxiety. TPM + BRV‐treated mice exhibited increased entries (8.000 ± 1.852; 113.3%) and time spent (92.463 ± 29.869 s; 196.3%) in the light chamber (*p* < 0.0001), with large effect sizes (*d* = 2.78 and 2.69, respectively). VPA, TMP, and BRV monotherapies showed no significant improvements (Figure 4a,b).

#### 3.3.4. EPM

The preference of mice to explore open and closed arms was evaluated in EPM to measure anxiety levels. 40‐PTZ mice spent minimal time (2.588 ± 2.379 s) and made few entries (1.125 ± 1.126) into open arms. TPM + BRV treatment significantly increased time spent (16.875 ± 6.770 s; 552%) and entries (6.875 ± 3.907; 511.1%) (*p* < 0.0001), with large effect sizes (*d* = 2.84 and 2.10, respectively). TMP and BRV alone showed no significant anxiolytic effects (Figure 4c,d).

#### 3.3.5. HBT

Mice′s anxious response toward exploring holes was evaluated in HBT by measuring latency to first head dip and total head dips. 40‐PTZ mice showed delayed initiation (4.188 ± 2.041 s) and reduced head dipping (59.250 ± 14.220). TPM + BRV‐treated mice demonstrated a 75.2% reduction in latency (1.038 ± 1.091 s) and a 66% increase in head dipping (93.375 ± 15.464; *p* < 0.0001), with large effect sizes (*d* = 1.98 and 2.43, respectively). VPA showed partial efficacy; TMP and BRV monotherapies were ineffective (Figure 4e,f).

#### 3.3.6. MBT

The tendency to bury marble evaluated in MBT and unburied marbles was used to assess compulsive‐like behavior. 40‐PTZ mice showed fewer unburied marbles (6.125 ± 2.031), while TPM + BRV‐treated mice left significantly more marbles unburied (10.250 ± 1.669; 67.3%, *p* = 0.0011), indicating reduced anxiety and compulsive behavior. The effect size was large (*d* = 2.27). VPA, TMP, and BRV monotherapies showed no significant effects (Figure 4g).

### 3.4. Memory and Learning Tests

#### 3.4.1. T Maze

**Figure 5:**
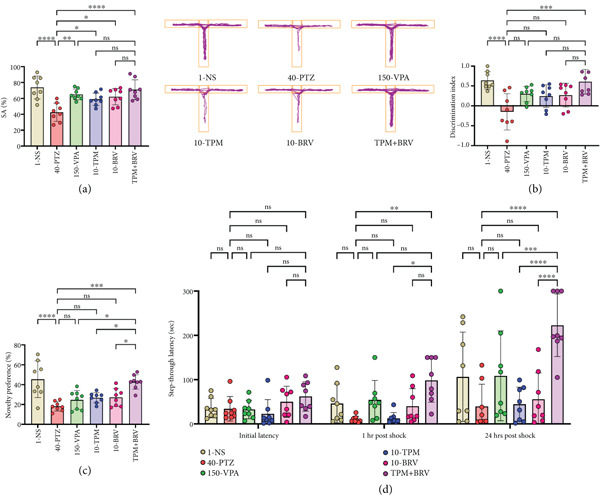
Treatment effect in (a) SA for T maze, (b) discrimination index in ORT, (c) % novelty preference in Y maze, and (d) the latency to enter the dark chamber for PAT. Significant effects were seen in the TPM + BRV group for all tests. All data were displayed as mean ± SD (*n* = 8), and statistical significance was  ^∗^ *p* < 0.05,  ^∗∗^ *p* < 0.01,  ^∗∗∗^ *p* < 0.001, and  ^∗∗∗∗^ *p* < 0.0001.

Spatial working memory in mice to remember the arm recently visited and exploration pattern, that is, SA behavior, was evaluated in the T maze. 40‐PTZ mice showed impaired performance (% SA: 42.563 ± 11.529), while TPM + BRV‐treated mice demonstrated a 66.8% improvement (% SA: 73.825 ± 14.475; *p* < 0.0001), with a large effect size (*d* = 2.70). VPA, TPM, and BRV monotherapies showed moderate significance but were less effective than the combination (Figure 5a).

#### 3.4.2. ORT

Learning and memory and the ability to differentiate between novel objects and previously familiarized objects were evaluated in ORT by measuring DI. 40‐PTZ mice failed to differentiate novel objects (DI: −0.149 ± 0.457), while TPM + BRV‐treated mice showed a 508.4% improvement (DI: 0.608 ± 0.311; *p* = 0.0002), with a large effect size (*d* = 2.74). VPA, TPM, and BRV monotherapies did not reach statistical significance (Figure 5b).

#### 3.4.3. Y Maze

Reference memory and long‐term memory were evaluated in the Y maze by measuring novelty preference. 40‐PTZ mice showed reduced novelty preference (18.213 ± 4.736), while TPM + BRV‐treated mice showed a 134.3% improvement (042.663 ± 7.285; *p* = 0.0002), with a large effect size (*d* = 3.95). Other treatments showed no significant improvement (Figure 5c)

#### 3.4.4. PAT

Behavior against an aversive stimulus was evaluated in PAT by measuring step‐through latency for short and long‐term memory retention. At 1 h, 40‐PTZ mice showed rapid entry into the aversive zone (10.113 ± 7.145 s), indicating poor short‐term memory. TPM + BRV‐treated mice exhibited an 873.8% increase in latency (98.488 ± 49.382 s; *p* = 0.0086), with a large effect size (*d* = 2.18), reflecting strong short‐term memory retention.

At 24 h, 40‐PTZ mice again showed impaired memory (latency: 39.525 ± 50.303 s). TPM + BRV‐treated mice demonstrated a 463.9% increase in latency (222.863 ± 70.744 s; *p* < 0.0001), with a large effect size (*d* = 2.75), indicating robust long‐term memory consolidation. Other treatments showed minimal or statistically insignificant improvements at both time points (Figure 5d).

#### 3.4.5. MWM

**Figure 6:**
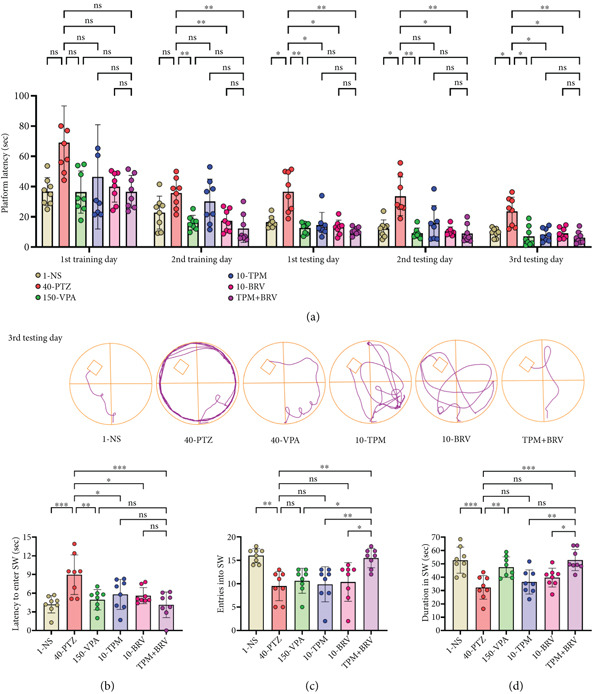
Treatment effect in MWM (a) escape latencies during five consecutive days (two training days and three testing days). For probe day (sixth day), (b) latency to the first entry in the SW zone, (c) entries, and (d) time duration in the SW zone. All data were displayed as mean ± SD (*n* = 8), and statistical significance was  ^∗^ *p* < 0.05,  ^∗∗^ *p* < 0.01, and  ^∗∗∗^ *p* < 0.001.

Long‐term working and reference memory were evaluated in MWM by measuring latency over 5 days. 40‐PTZ mice showed prolonged escape latencies across all sessions, with the longest latency on Day 5 (23.550 ± 9.430 s), indicating impaired spatial learning. TPM + BRV‐treated mice showed the shortest latency (6.325 ± 3.987 s; *p* = 0.0084), reflecting a 73.1% reduction in escape time compared to PTZ‐kindled mice, with a large effect size (*d* = 2.35). All treatment groups showed gradual improvement, but TPM + BRV consistently outperformed the others (Figure 6a).

On probe day, the platform was removed from the SW zone, and mice were evaluated for long‐term learning and memory capability to remember platform location and time swimming in the SW zone. Parameters were latency to first entry, entries, and time spent in the SW zone. 40‐PTZ mice showed impaired recall (latency: 0.975 ± 3.186 s; entries: 9.500 ± 3.117; time: 32.300 ± 8.822 s). TPM + BRV‐treated mice demonstrated a 53.9% reduction in latency, a 63.2% increase in entries, and a 63.5% increase in time spent in the target zone (*p* = 0.0002–0.0035), with large effect sizes (*d* = 2.01–2.58). While VPA, TPM, and BRV monotherapies improved latency, they failed to enhance entries or time spent in the target zone significantly. TPM + BRV showed the most robust memory retention across all parameters (Figures 6b, 6c, and 6d).

### 3.5. Depressive‐Like Behavior Tests

#### 3.5.1. SPT

Anhedonia and depressive‐like behavior through sweet solution preference were evaluated in SPT. 40‐PTZ mice showed reduced preference (37.438 ± 9.597), indicating depressive‐like behavior. TPM + BRV‐treated mice demonstrated a 96.7% increase in sucrose preference (74.304 ± 18.643; *p* = 0.0002), with a large effect size (*d* = 3.91). VPA, TPM, and BRV monotherapies showed no significant improvement (Figure 7a).

Figure 7Treatment effect in depressive‐like tests: (a) % sucrose preference in SPT, (b) immobility time in FST, and (c) immobility time in TST. All data were displayed as mean ± SD (*n* = 8), and statistical significance was  ^∗^ *p* < 0.05,  ^∗∗^ *p* < 0.01,  ^∗∗∗^ *p* < 0.001, and  ^∗∗∗∗^ *p* < 0.0001.

**Figure:**
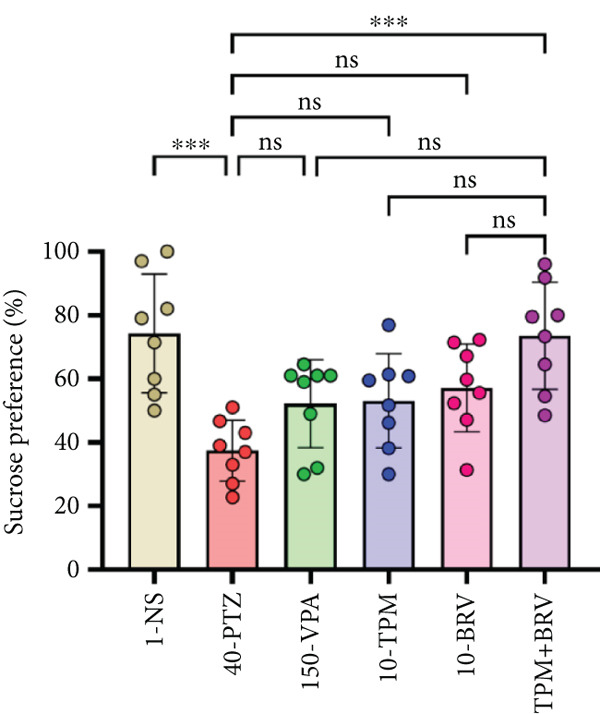
(a)

**Figure:**
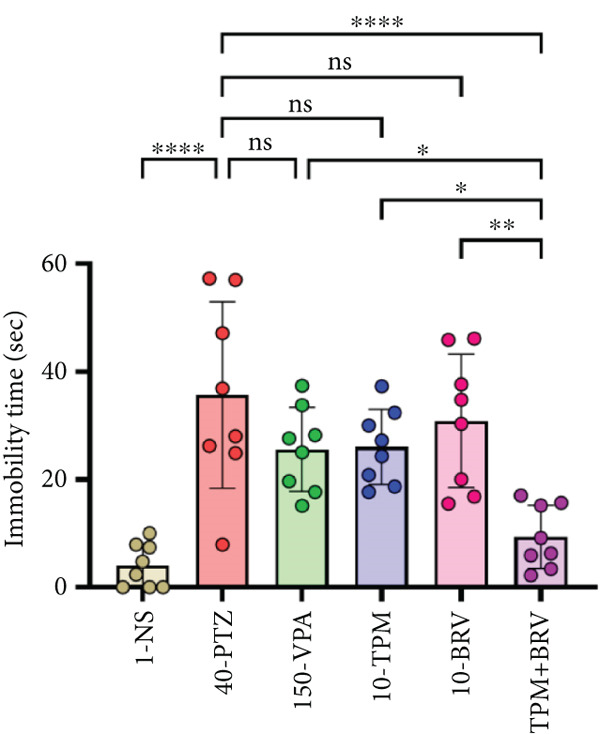
(b)

**Figure:**
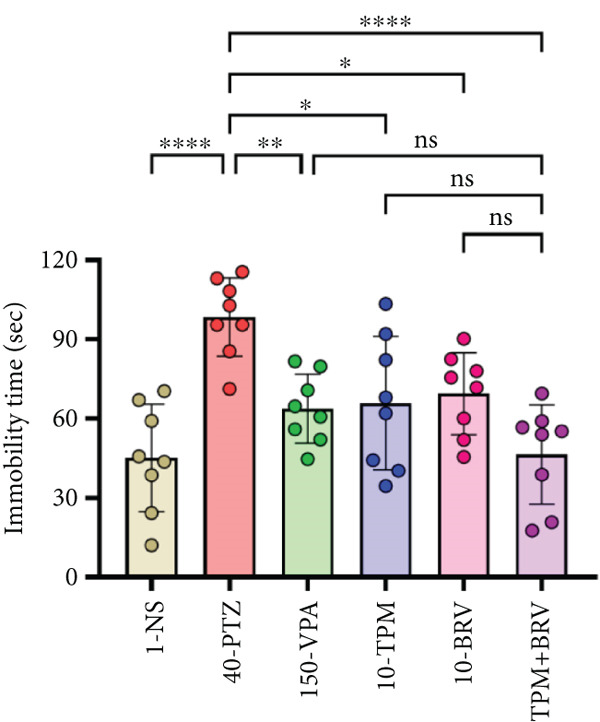
(c)

#### 3.5.2. FST

Behavioral despair was evaluated in FST by measuring immobility time. 40‐PTZ mice showed the highest immobility time (35.663 ± 17.282 s), while TPM + BRV exhibited a 73.7% reduction (4.063 ± 4.034 s; *p* < 0.0001), with a large effect size (*d* = 2.05). Other treatments failed to produce significant reductions (Figure 7b).

#### 3.5.3. TST

Depressive‐like behavior through immobility time was evaluated in TST. 40‐PTZ mice showed prolonged immobility time (98.425 ± 14.823 s), while TPM + BRV showed a 52.7% reduction (45.163 ± 20.267 s; *p* < 0.0001), with a large effect size (*d* = 1.37). PVA, TPM, and BRV monotherapies showed moderate significance but were less effective than the combination (Figure 7c).

#### 3.5.4. Treatment Effect on Neurochemical Analysis

Oxidative stress induced by PTZ kindling was evaluated using an MDA assay. 40‐PTZ mice showed elevated lipid peroxidation (1085 ± 127.745 nmol/mg), while TPM + BRV‐treated mice exhibited a 70.7% reduction (317.658 ± 34.877 nmol/mg; *p* = 0.0005), with a large effect size (*d* = 7.78). VPA and BRV monotherapies showed moderate reductions; TPM was not significant (Figure 8a).

Figure 8Treatment effect in neurochemical assays after kindling process (*n* = 4). (a) MDA, (b) SOD, (c) GPx, (d) CAT, and (e) AChE. All data were displayed as mean ± SD (*n* = 4), and statistical significance was  ^∗^ *p* < 0.05,  ^∗∗^ *p* < 0.01,  ^∗∗∗^ *p* < 0.001, and  ^∗∗∗∗^ *p* < 0.0001.

**Figure:**
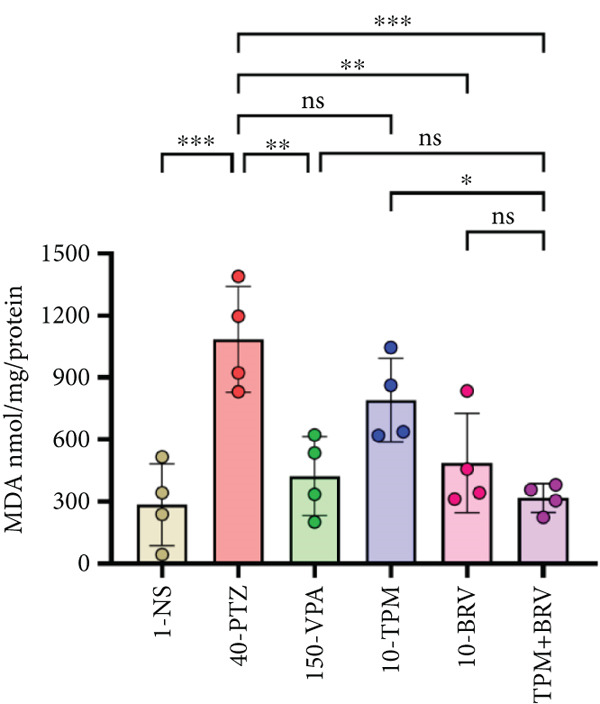
(a)

**Figure:**
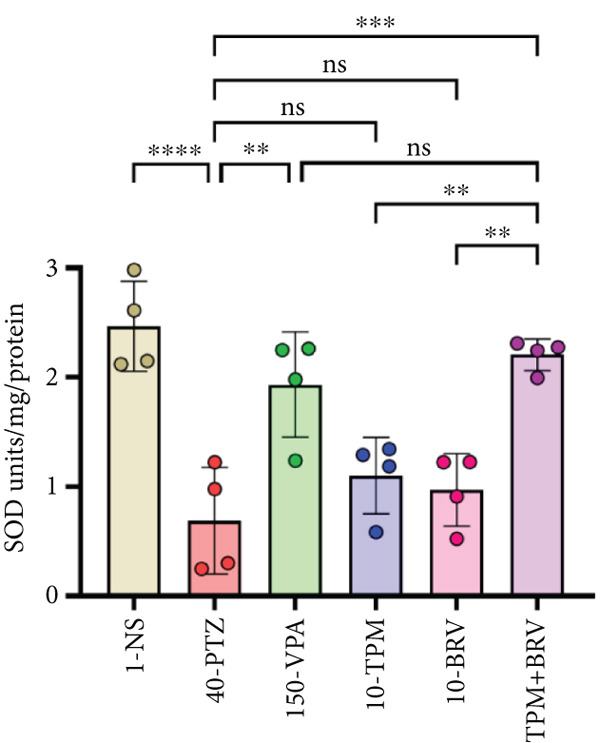
(b)

**Figure:**
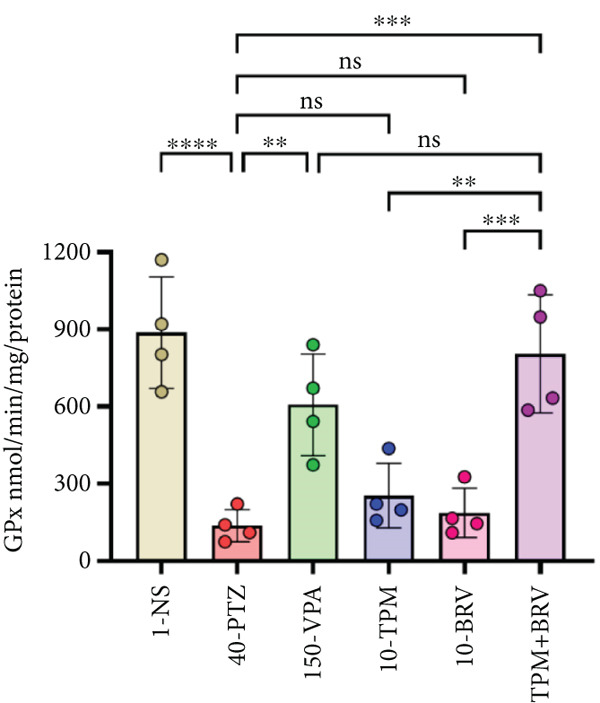
(c)

**Figure:**
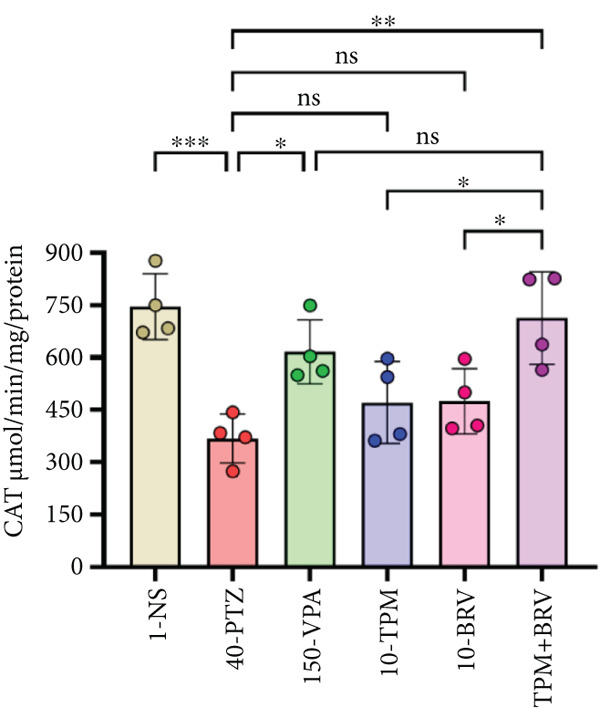
(d)

**Figure:**
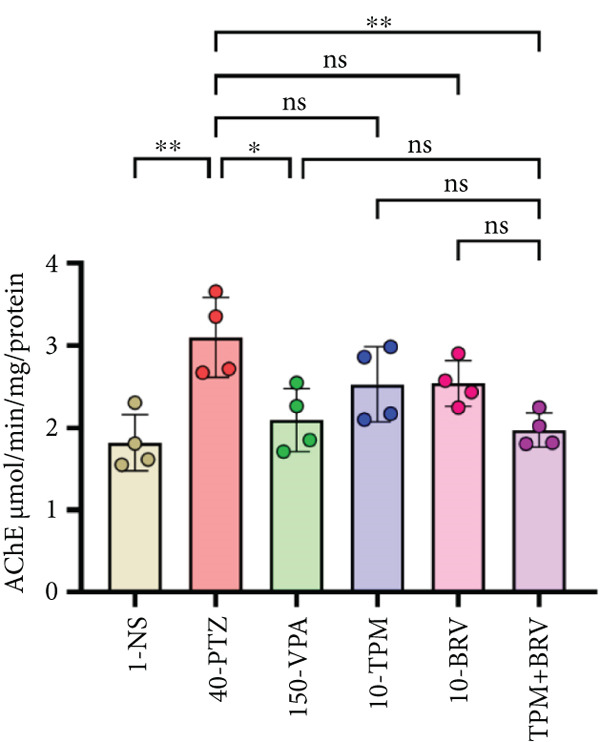
(e)

Moreover, the neuroprotective, anti‐inflammatory, and antioxidant functions were evaluated using the SOD assay. 40‐PTZ mice showed elevated lipid peroxidation (*μ*/mg), indicating impaired antioxidant defense. TPM + BRV‐treated mice exhibited a 220% increase in SOD levels (2.208 ± 0.144 * μ*/mg; *p* = 0.0003), with a very large effect size (*d* = 3.79). VPA also showed significant improvement, while TMP and BRV monotherapies were ineffective. TPM + BRV was significantly more effective than TPM alone (*p* = 0.0082) and BRV alone (*p* = 0.0030) (Figure 8b).

Reactive oxygen species reactions are terminated by several endogenous antioxidant enzymes such as GPx. Free radical accumulation resulted in neuronal oxidative stress observed with chemoconvulsants such as PTZ. GPx levels were lowest in 40‐PTZ mice (136.602 ± 62.458 nmol/min/mg), reflecting oxidative stress and neuronal damage. TPM + BRV‐treated mice exhibited a 489.2% increase in GPx levels (804.872 ± 229.405 nmol/min/mg; *p* = 0.0003), with a very large effect size (*d* = 3.78). VPA also showed a significant elevation, while TMP and BRV monotherapies were not significant. TPM + BRV was more effective than TPM alone (*p* = 0.0023) and BRV alone (*p* = 0.0007) (Figure 8c).

CAT, which acts as an endogenous antioxidant defense, was evaluated. CAT levels were lowest in 40‐PTZ mice (368.101 ± 69.837 * μ*mol/min/mg), indicating weakened antioxidant defense. TPM + BRV‐treated mice showed a 93.6% increase in CAT levels (712.709 ± 132.482 * μ*mol/min/mg; *p* = 0.0017), with a large effect size (*d* = 2.91). VPA also showed significant improvement, while TMP and BRV monotherapies were not significant. TPM + BRV was more effective than TPM alone (*p* = 0.0346) and BRV alone (*p* = 0.0385) (Figure 8d).

Additionally, AChE activity was also evaluated, and 40‐PTZ mice showed elevated AChE levels (3.101 ± 0.486 * μ*mol/min/mg), indicating cholinergic dysfunction. TPM + BRV‐treated mice demonstrated a 36.4% reduction (1.973 ± 0.0.208 * μ*mol/min/mg; *p* = 0.0050), with a large effect size (*d* = 2.25). VPA showed moderate efficacy; TMP and BRV monotherapies were not significant (Figure 8e).

#### 3.5.5. Integrative Summary

In all neurobehavioral tests, combination therapy consistently displayed robust improvements in all behavioral parameters with large to very large effect sizes (Cohen′s *d* = 1.73–4.76). These behavioral improvements were accompanied by neurobiochemical improvements, such as decreased lipid peroxidation (71.7% MDA), increased antioxidant enzyme activity (220.9% SOD, 489.2% GPx, and 93.6% CAT), and restored cholinergic tone (36.4% AChE). The magnitude and stability of TPM + BRV effects indicate a synergistic action that alleviates oxidative stress and neuroinflammation induced by PTZ, thereby restoring functional performance. This neurobehavioral and neurobiochemical recovery link effect shows TPM + BRV as a potential therapeutic approach to epilepsy‐associated neuropsychiatric impairments.

## 4. Discussion

Although over 30 AEDs are currently available, epilepsy resistant to drug treatment and neuropsychiatric adverse effects due to its treatment elicit great clinical concern. The logic behind polytherapy, especially a low‐dose regimen, has recently formed a potential option in enhancing seizure control without much adverse effect [7]. In this respect, the current study explored the effectiveness of coadministering a low dose of TPM and BRV in the PTZ‐kindled mouse model.

Combination therapy of TPM and BRV protected all mice against tonic–clonic seizures induced by repeated PTZ doses, whereas monotherapy gradually showed severe seizures during the kindling process, consistent with our previous findings [14]. We found that the combination of the two drugs in low doses resulted in significantly better outcomes, concerning all examined parameters of both behavior and biochemistries, compared to either drug alone, which showed only modest improvements. Cotreated mice demonstrated improved memory, reduced anxiety and depressive‐like behavior, and enhanced antioxidant systems, as evidenced by normalized SOD, CAT, and GPx and lowered MDA and AChE activity. These effects may result from synergetic interactions of TPM, which increases GABAergic inhibition and decreases the excitatory neurotransmission levels, and BRV, which interacts with SV2A‐mediated release of synaptic vesicles. Although these mechanisms have been reinforced by the previous literature, they are still hypothetical and need to be validated mechanistically. Notably, the improvements in observed behaviors are consistent with clinical issues, as patients with epilepsy often experience cognitive decline, anxiety, and depression, which are not only worsened by seizures but also by some antiseizure medications [1]. The present results demonstrate the promise of low‐dose rational polytherapy to not only control seizures but also minimize pharmacoresistance and mitigate long‐term neuropsychiatric comorbidities.

PTZ kindling is a chronic model of experimental epilepsy that offers opportunities to study a range of progressive comorbid conditions associated with epilepsy. Approximately 45% of epileptic patients experience anxiety, which negatively affects their quality of life [40]. The PTZ‐treated mice showed marked reductions in exploration of central, lighted, novel, and elevated zones in OFT, LDT, SIT, and EPM, indicating a strong anxiogenic‐like effect, possibly by GABA_A_ receptor antagonism [41]. Therapy with BRV, TPM, and particularly the combination reduced these anxiety‐like behaviors through the demonstration of increased exploration of open and elevated areas, increased hole‐picking in HBT, and decreased marble burying in MBT. Social anxiety was also reduced, and the treated mice became more interactive in SIT. TPM + BRV and BRV alleviated locomotor impairments in PTZ‐kindled mice, which indicates lowered anxiety‐induced hypoactivity. Our findings align with previous preclinical studies that reported anxiolytic effects of TPM and BRV [42].

Epilepsy‐related depression using TST, FST, and SPT was evaluated in the PTZ kindling model. Depressive‐like behaviors, that is, increased immobility and reduced sucrose intake, were noted in PTZ‐kindled mice, while these alterations were mitigated by monotherapy with TPM and BRV but were more prominent with their combination. These findings are supported by previously reported antidepressant‐like effects of TPM [43] and BRV [11], therefore highlighting the potential of the combination as an effective therapy for preventing neuropsychiatric symptoms.

Furthermore, cognitive deficits induced by PTZ kindling were apparent across spatial, recognition, and novelty‐based memory. PTZ‐treated mice showed decreased % SA in the T maze, reduced DI in ORT, and lower novelty preference in the Y maze test. In the MWM test, the PTZ‐kindled mice exhibited increased escape latency and reduced platform zone entries and time spent. Monotherapy with TPM and BRV partially reversed these deficits, while the combination of both drugs restored cognitive performance throughout all cognitive tests. These findings are consistent with studies that reported the memory‐improving potential of BRV and TPM. TPM treatment alleviated the PTZ‐induced convulsions in rats and was associated with a significant improvement in behavioral alterations, brain mediators, and oxidative stress in hippocampal regions [44]. Shishmanova‐Doseva [45] observed improvement in learning abilities in both active and PATs with TPM treatment. Farooq et al. [11] reported remarkable improvements in animal anxiety and cognition with BRV treatment. In contrast, Agarwal et al. [10] showed that treatment with TPM alone impaired cognition, while Zwierzyńska and Pietrzak [46] concluded that BRV might cause anxiety disturbance and transient memory deficits. Such inconsistencies may be attributed to variations in dose, treatment duration, species, and behavioral testing protocols, emphasizing the need for cautious interpretation.

The main key in the pathophysiology of epilepsy is oxidative stress, which causes neuronal degeneration and comorbid symptoms. PTZ‐kindled mice demonstrated lower levels of SOD, GPx, and CAT and elevated MDA and AChE levels. These findings are consistent with previously reported studies [47, 48]. Treatment with combined TPM and BRV reversed these changes, suggesting an antioxidant restoration function. Monotherapy effects were noted but were not as significant. These neurobiochemical enhancements may be linked to the observed neurobehavioral recovery.

### 4.1. Limitations and Future Directions

This study has several limitations, including small sample sizes for neurobiochemical assays, the use of male‐only animals, a single dose, and a single seizure model, which may restrain universality. Future studies should include assessments of dose‐response, multiple seizure models, and sex differences. Mechanistic studies are also needed to verify the hypothesized interactions between TPM and BRV.

## 5. Conclusion

In conclusion, the combination of TPM plus BRV was more effective in improving seizure suppression, neurobehavioral impairments, and oxidative stress indicators in PTZ‐kindled mice than when used as monotherapies. Although each agent alone showed modest neuroprotective and behavioral effects, their combined treatment was associated with enhanced outcomes in anxiety, memory, and redox balance, suggesting a potential synergistic interaction. These findings emphasize the impact of cognitive and psychiatric comorbidities associated with epilepsy and suggest that rational polytherapy strategies may propose a promising approach to alleviate these deficits. However, certain study limitations should be elucidated with caution, including small sample sizes for neurobiochemical assays, the use of male‐only animals, a single dose, and a single seizure model. Further research is warranted to assess the long‐term safety, dose‐dependent efficacy, sex differences, and clinical relevance of this combination across diverse epilepsy models.

## Ethics Statement

Ethical approval was obtained from the Departmental Research Ethical Committee for the utilization of laboratory animals (Approval No. 02/PHDL/S/23, 07/05/2024) of the Department of Pharmacology, B.Z. University, Multan.

## Disclosure

All authors read and approved the final manuscript.

## Conflicts of Interest

The authors declare no conflicts of interest.

## Author Contributions

Methodology, software, investigation, writing—original draft, writing—review and editing, visualization, conceptualization: K.A.S. Methodology, validation, writing—original draft, writing—review and editing: W.A. Validation, data curation, writing—review and editing: R.M.Z.M. Conceptualization, resources, writing—original draft, writing—review and editing, supervision, funding acquisition, project administration: F.A. Conceptualization, resources, writing—original draft, writing—review and editing, supervision, visualization: I.I.

## Funding

This work was supported by the Ongoing Research Funding Programme (ORF‐2025‐131) King Saud University, Riyadh, Saudi Arabia.

## Supporting information


**Supporting Information** Additional supporting information can be found online in the Supporting Information section. Table S1. Summary of all test parameters with *F* and *p* values from statistical analyses. Table S2. Relative improvements and effect sizes of VPA, TPM, BRV, and TPM + BRV compared to PTZ‐kindled mice for all test parameters.

## Data Availability

The datasets used and/or analyzed during the current study are available from the corresponding authors upon reasonable request.
