# Supplementary material for: Brivaracetam Combined With Topiramate at Low Doses Alleviates Neurobehavioral Deficits and Oxidative Stress in a Chemoconvulsant Kindled Seizure Model
Source: Behav Neurol. 2025 Dec 11;2025:8037864. doi: 10.1155/bn/8037864 (PMC12698180; doi:10.1155/bn/8037864)
Supplement: Supplementary file 1 — Supporting Information Additional supporting information can be found online in the Supporting Information section. Table S1. Summary of all test parameters with F and p values from statistical analyses. Table S2. Relative improvements and effect sizes of VPA, TPM, BRV, and TPM + BRV compared to PTZ‐kindled mice for all test parameters. [file BN-2025-8037864-s001.docx]

**Brivaracetam combined with topiramate at low doses alleviates neurobehavioral deficits and oxidative stress in a chemoconvulsant kindled seizure model**

Khaled Ahmed Saghir^1^, Waseem Ashraf ^1^, Rana Muhammad Zahid Mushtaq^2^, Faleh Alqahtani^3^, Imran Imran^1*^

1 Department of Pharmacology, Faculty of Pharmacy, Bahauddin Zakariya University, Multan 60800, Pakistan.

2 Institute for Regeneration and Repair, Edinburgh Medical School, The University of Edinburgh, United Kingdom.

3 Department of Pharmacology and Toxicology, College of Pharmacy, King Saud University, Riyadh 11451, Saudi Arabia.

* Correspondence: *Dr. Imran Imran, Department of Pharmacology, Faculty of Pharmacy, Bahauddin Zakariya University 60800, Multan, Pakistan. E-mail: imran.ch@bzu.edu.pk; Tel: +92- 61-9210465; Fax: +92-61-9210098.

*Dr. Faleh Alqahtani, Department of Pharmacology and Toxicology, College of Pharmacy, King Saud University, Riyadh, Saudi Arabia. E-mail: afaleh@ksu.edu.sa

**Table S1.** Summary of all test parameters with F and P values

| Test | Parameter | F value (df) | P value |
| --- | --- | --- | --- |
| Anxiety-like behavior tests | | | |
| Open Field Test | Distance traveled | F(5,42) = 10.47 | P < 0.0001 |
|  | Speed | F(5,42) = 9.57 | P < 0.0001 |
|  | Time in central zone | F(5,42) = 10.25 | P < 0.0001 |
|  | Entries into central zone | F(5,42) = 13.25 | P < 0.0001 |
|  | Fast rearings | F(5,42) = 8.611 | P < 0.0001 |
|  | Fast movements | F(5,42) = 10.33 | P < 0.0001 |
| Light/Dark Test | Entries into light chamber | F(5,42) = 10.68 | P < 0.0001 |
|  | Time spent in the light chamber | F(5,42) = 12.21 | P < 0.0001 |
| Elevated Plus Maze | Time in open arms | F(5,42) = 16.69 | P < 0.0001 |
|  | Entries into open arms | F(5,42) = 10.10 | P < 0.0001 |
| Hole Board Test | Latency to first head dip | F(5,42) = 5.774 | P = 0.0004 |
|  | Number of head dips | F(5,42) = 6.763 | P = 0.0001 |
| Marble Burying Test | Number of unburied marbles | F(5,42) = 12.61 | P < 0.0001 |
| Memory and learning tests | | | |
| T Maze | % Spontaneous Alternation | F(5,42) = 8.354 | P < 0.0001 |
| Object Recognition Test | Discrimination Index (DI) | F(5,42) = 7.075 | P < 0.0001 |
| Y Maze | % Novelty Preference | F(5,42) = 9.130 | P < 0.0001 |
| Passive Avoidance Test | Step-through latency (1 h) | F(5,42) = 12.89 | P < 0.0001 |
|  | Step-through latency (24 h) | F(5,42) = 12.89 | P < 0.0001 |
| Morris Water Maze | Escape latency (Day 1–5) | F(5,42) = 20.20 | P < 0.0001 |
|  | Latency to first entry (SW zone) | F(5,42) = 5.758 | P = 0.0004 |
|  | Entries into the SW zone | F(5,42) = 7.648 | P < 0.0001 |
|  | Time spent in the SW zone | F(5,42) = 8.499 | P < 0.0001 |
| Depressive-like behavior tests | | | |
| Sucrose Preference Test | Sucrose intake | F(5,42) = 7.166 | P < 0.0001 |
| Forced Swim Test | Immobility time | F(5,42) = 12.18 | P < 0.0001 |
| Tail Suspension Test | Immobility time | F(5,42) = 8.852 | P < 0.0001 |
| Neurochemical assays | | | |
| MDA | Lipid peroxidation (nmol/mg) | F(5,18) = 9.590 | P < 0.0001 |
| SOD | Superoxide dismutase (u/mg) | F(5,18) = 14.53 | P < 0.0001 |
| GPx | Glutathione peroxidase (nmol/mg) | F(5,18) = 15.55 | P < 0.0001 |
| CAT | Catalase (µmol/min/mg) | F(5,18) = 8.718 | P = 0.0002 |
| AChE | Acetylcholinesterase (µmol/min/mg) | F(5,18) = 6.480 | P = 0.0013 |

**Table S2.** Relative improvements and effect sizes of VPA, TPM, BRV, and TPM+BRV vs PTZ-kindled mice for all test parameters. Absolute effect sizes were considered to be very small (d = 0.01), small (d = 0.20), medium (d = 0.50), large (d = 0.80), and very large (d ≥ 1.30). Positive values represent improvement, while negative values demonstrate beneficial reduction.

| Test | Parameter | Group | Relative Improvement (%) | Effect Size (Cohen’s d) | Interpretation |
| --- | --- | --- | --- | --- | --- |
| PTZ-kindling | **Seizure score** | VPA | -60.5% | -4.11 | Very large (reduction) |
|  |  | TPM | -20.9% | -1.37 | Very large (reduction) |
|  |  | BRV | -25.6% | -1.83 | Very large (reduction) |
|  |  | **TPM+BRV** | **-88.4%** | **-4.79** | **Very large (reduction)** |
| Anxiety-like behavior tests | | | | | |
| OFT | **Distance traveled** | VPA | +99.6% | +1.78 | Very large |
|  |  | TPM | +84.3% | +1.31 | Very large |
|  |  | BRV | +106.4% | +1.70 | Very large |
|  |  | **TPM+BRV** | **+223.8%** | **+4.72** | **Very large** |
|  | **Speed** | VPA | +82.0% | +1.37 | Very large |
|  |  | TPM | +72.0% | +1.74 | Very large |
|  |  | BRV | +92.0% | +1.67 | Very large |
|  |  | **TPM+BRV** | **+186.0%** | **+3.45** | **Very large** |
|  | **Time in central zone (s)** | VPA | +62.9% | +1.59 | Very large |
|  |  | TPM | +27.4% | +0.63 | Medium |
|  |  | BRV | +52.4% | +1.19 | Large |
|  |  | **TPM+BRV** | **+151.8%** | **+3.02** | **Very large** |
|  | **Entries into central zone** | VPA | +136.8% | +2.31 | Very large |
|  |  | TPM | +57.9% | +1.06 | Very large |
|  |  | BRV | +154.4% | +1.78 | Very large |
|  |  | **TPM+BRV** | **+259.6%** | **+3.87** | **Very large** |
|  | **Fast Rearing** | VPA | +58.5% | +0.77 | Large |
|  |  | TPM | +83.0% | +1.28 | Very large |
|  |  | BRV | +128.3% | +2.42 | Very large |
|  |  | **TPM+BRV** | **+173.6%** | **+5.25** | **Very large** |
|  | **Fast Movements** | VPA | +29.1% | +0.87 | Large |
|  |  | TPM | +23.2% | +0.83 | Large |
|  |  | BRV | +41.3% | +1.53 | Very large |
|  |  | **TPM+BRV** | **+79.3%** | **+4.76** | **Very large** |
| SIT | **Sociability Index** | VPA | +162.2% | +2.86 | Very large |
|  |  | TPM | +62.7% | +1.06 | Very large |
|  |  | BRV | +74.3% | +1.33 | Very large |
|  |  | **TPM+BRV** | **+195.2%** | **+3.56** | **Very large** |
|  | **Social novelty preference (%)** | VPA | +34.3% | +1.43 | Very large |
|  |  | TPM | +4.2% | +0.17 | Very small |
|  |  | BRV | +6.6% | +0.27 | Small |
|  |  | **TPM+BRV** | **+71.0%** | **+2.73** | **Very large** |
| LDT | **Entries into the light chamber** | VPA | +53.3% | +1.68 | Very large |
|  |  | TPM | +36.7% | +1.13 | Very large |
|  |  | BRV | +50.0% | +1.37 | Very large |
|  |  | **TPM+BRV** | **+113.3%** | **+2.78** | **Very large** |
|  | **Time in the light chamber (S)** | **VPA** | **+99.3%** | **+2.56** | **Very large** |
|  |  | TPM | +70.0% | +1.31 | Very large |
|  |  | BRV | +77.0% | +1.33 | Very large |
|  |  | **TPM+BRV** | **+196.3%** | **+2.69** | **Very large** |
| EPM | **Time in open arms (s)** | VPA | +355.9% | +3.40 | Very large |
|  |  | TPM | +64.2% | +0.66 | Medium |
|  |  | BRV | +120.2% | +0.89 | Large |
|  |  | **TPM+BRV** | **+552.0%** | **+2.84** | **Very large** |
|  | **Entries into open arms** | VPA | +311.1% | +2.16 | Very large |
|  |  | TPM | +100.0% | +0.59 | Medium |
|  |  | BRV | +44.4% | +0.47 | Small |
|  |  | **TPM+BRV** | **+511.1%** | **+2.10** | **Very large** |
| HBT | **latency to 1st head dip**  **(s)** | VPA | -67.5% | -1.82 | Very Large (reduction) |
|  |  | TPM | -50.7% | -1.21 | Very Large (reduction) |
|  |  | BRV | -63.3% | -1.65 | Very Large (reduction) |
|  |  | **TPM+BRV** | **-75.2%** | **-1.98** | **Very Large (reduction)** |
|  | **Head dips** | VPA | +31.4% | +1.41 | Very large |
|  |  | TPM | +16.2% | +0.72 | Medium |
|  |  | BRV | +31.6% | +1.27 | Very large |
|  |  | **TPM+BRV** | **+66.0%** | **+2.43** | **Very large** |
| MBT | **Unburied marbles** | VPA | +36.7% | +1.24 | Very large (increase) |
|  |  | TPM | -8.2% | -0.27 | Small (reduction) |
|  |  | BRV | -18.4% | -0.63 | Medium (reduction) |
|  |  | **TPM+BRV** | **+67.3%** | **+2.27** | **Very large (increase)** |
| Memory and learning tests | | | | | |
| T Maze | **Spontaneous Alternation (%)** | VPA | +52.6% | +2.37 | Very large |
|  |  | TPM | +38.5% | +1.67 | Very large |
|  |  | BRV | +46.0% | +1.97 | Very large |
|  |  | **TPM+BRV** | **+66.8%** | **+2.70** | **Very large** |
| ORT | **Discrimination Index (DI)** | VPA | +301.7% | +1.27 | Large |
|  |  | TPM | +265.5% | +1.09 | Large |
|  |  | BRV | +281.5% | +1.21 | Large |
|  |  | **TPM+BRV** | **+508.4%** | **+2.74** | **Very large** |
| Y Maze | **Novelty Preference (%)** | VPA | +35.3% | +0.85 | Large |
|  |  | TPM | +47.3% | +1.87 | Very large |
|  |  | BRV | +49.0% | +1.26 | Large |
|  |  | **TPM+BRV** | **+134.3%** | **+3.95** | **Very large** |
| PAT | **1-hour post-shock**  **Latency (s)** | VPA | +435.0% | +1.36 | Very large |
|  |  | TPM | +24.3% | +0.12 | Very small |
|  |  | BRV | +294.8% | +0.92 | Large |
|  |  | **TPM+BRV** | **+873.8%** | **+2.18** | **Very large** |
|  | **24-hour post-shock**  **Latency (s)** | VPA | +175.1% | +0.87 | Large |
|  |  | TPM | +12.7% | +0.06 | Very small |
|  |  | BRV | +40.9% | +0.18 | Very small |
|  |  | **TPM+BRV** | **+463.9%** | **+2.75** | **Very large** |
| MWM | **1st training day**  **Latency (s)** | VPA | -47.4% | -1.67 | Very large (reduction) |
|  |  | TPM | -32.7% | -0.82 | Large (reduction) |
|  |  | BRV | -42.3% | -1.60 | Very large (reduction) |
|  |  | **TPM+BRV** | **-47.1%** | **-1.73** | **Very large (reduction)** |
|  | **2nd training day**  **Latency (s)** | VPA | -54.5% | -2.57 | Very large (reduction) |
|  |  | TPM | -15.3% | -0.46 | Small (reduction) |
|  |  | BRV | -51.1% | -2.05 | Very large (reduction) |
|  |  | **TPM+BRV** | **-65.4%** | **-2.80** | **Very large (reduction)** |
|  | **1st testing day**  **Latency (s)** | VPA | -65.4% | -2.51 | Very large (reduction) |
|  |  | TPM | -60.1% | -2.37 | Very large (reduction) |
|  |  | BRV | -64.1% | -2.60 | Very large (reduction) |
|  |  | **TPM+BRV** | **-70.6%** | **-2.87** | **Very large (reduction)** |
|  | **2nd testing day**  **Latency (s)** | VPA | -72.4% | -2.62 | Very large (reduction) |
|  |  | TPM | -52.7% | -1.64 | Very large (reduction) |
|  |  | BRV | -68.7% | -2.48 | Very large (reduction) |
|  |  | **TPM+BRV** | **-73.3%** | **-2.44** | **Very large (reduction)** |
|  | **3rd testing day**  **Latency (s)** | VPA | -69.7% | -2.12 | Very large |
|  |  | TPM | -64.8% | -2.11 | Very large |
|  |  | BRV | -60.7% | -1.96 | Very large |
|  |  | **TPM+BRV** | **-73.1%** | **-2.35** | **Very large** |
|  | **Probe day: Latency to SW (s)** | VPA | -44.8% | -1.63 | Very large (reduction) |
|  |  | TPM | -35.2% | -1.46 | Very large (reduction) |
|  |  | BRV | -37.5% | -1.55 | Very large (reduction) |
|  |  | **TPM+BRV** | **-53.9%** | **-2.01** | **Very large (reduction)** |
|  | **Probe day: Entries into SW** | VPA | +11.8% | +0.38 | Small |
|  |  | TPM | +3.9% | +0.10 | Very small |
|  |  | BRV | +9.2% | +0.24 | Small |
|  |  | **TPM+BRV** | **+63.2%** | **+2.13** | **Very large** |
|  | **Probe day: Duration in SW (s)** | VPA | +47.1% | +1.87 | Very large |
|  |  | TPM | +12.7% | +0.49 | Small |
|  |  | BRV | +22.8% | +0.94 | Large |
|  |  | **TPM+BRV** | **+63.5%** | **+2.58** | **Very large** |
| Depressive-like behavior tests | | | | | |
| SPT | **Sucrose preference (%)** | VPA | +39.4% | +1.20 | Large |
|  |  | TPM | +41.9% | +1.47 | Very Large |
|  |  | BRV | +52.7% | +1.59 | Very Large |
|  |  | **TPM+BRV** | **+96.7%** | **+3.91** | **Very Large** |
| FST | **Immobility time (s)** | VPA | –28.3% | –0.75 | **Medium (reduction)** |
|  |  | TPM | –29.7% | –0.74 | **Medium (reduction)** |
|  |  | BRV | –13.4% | –0.43 | **Small (reduction)** |
|  |  | **TPM+BRV** | **–73.7%** | **–2.05** | **Very Large (reduction)** |
| TST | **Immobility time (s)** | VPA | –35.3% | –0.91 | **Large (reduction)** |
|  |  | TPM | –32.0% | –0.91 | **Large (reduction)** |
|  |  | BRV | –29.4% | –0.76 | **Medium (reduction)** |
|  |  | **TPM+BRV** | **–52.7%** | **–1.37** | **Very Large (reduction)** |
| Neurochemical assays | | | | | |
| MDA | **(nmol/mg/protein)** | VPA | –60.9% | –5.86 | **Very Large (reduction)** |
|  |  | TPM | –30.2% | –2.63 | **Very Large (reduction)** |
|  |  | BRV | –54.2% | –4.93 | **Very Large (reduction)** |
|  |  | **TPM+BRV** | **–70.7%** | **–7.78** | **Very Large (reduction)** |
| SOD | **(Unit/mg/protein** | VPA | +108.4% | +1.71 | **Very Large** |
|  |  | TPM | +60.0% | +0.96 | **Large** |
|  |  | BRV | +41.3% | +0.70 | **Medium** |
|  |  | **TPM+BRV** | **+220.9%** | **+3.79** | **Very Large** |
| GPx | **(nmol/min/mg/protein)** | 150-VPA | +344.4% | +2.96 | Very large |
|  |  | 10-TPM | +85.9% | +1.08 | Very large |
|  |  | 10-BRV | +36.5% | +0.56 | Medium |
|  |  | **TPM+BRV** | **+489.2%** | **+3.78** | **Very large** |
| CAT | **(µmol/min/mg/protein)** | VPA | +70.0% | +3.08 | **Very Large** |
|  |  | TPM | +27.9% | +0.84 | **Large** |
|  |  | BRV | +29.0% | +1.16 | **Very Large** |
|  |  | **TPM+BRV** | **+93.6%** | **+2.91** | **Very Large** |
| AChE | **(µmol/min/mg/protein)** | VPA | –32.5% | –2.37 | Very Large (reduction) |
|  |  | TPM | –18.4% | –1. 24 | Large (reduction) |
|  |  | BRV | –18.1% | –1.42 | Very Large (reduction) |
|  |  | **TPM+BRV** | **–36.4%** | **–2.45** | **Very Large (reduction)** |
